# Preoperative frailty risk in older patients with colorectal cancer admitted to the ICU after surgery: prevalence, associated factors, and association with in-hospital outcomes

**DOI:** 10.3389/fonc.2026.1948607

**Published:** 2026-10-26

**Authors:** Yongqin Wang, Guifang Li, Qiuyan Yang, Meng Yin, Xingyu Yang, Jiaying Li

**Affiliations:** 1 School of Nursing, Ningxia Medical University, Yinchuan, China; 2 Department of Trauma Orthopedics, General Hospital of Ningxia Medical University, Yinchuan, China; 3 Department of Critical Care Medicine, Cardio-cerebrovascular Disease Hospital, General Hospital of Ningxia Medical University, Yinchuan, China; 4 Department of First Department of Surgical Oncology, General Hospital of Ningxia Medical University, Yinchuan, China; 5 Department of Critical Care Medicine, Sun Yat-sen Memorial Hospital, Sun Yat-sen University, Guangzhou, Guangdong, China

**Keywords:** frailty, intensive care unit, colorectal cancer surgery, older adults, postoperative complications, associated factors, MFI-5

## Abstract

**Introduction:**

Frailty is recognized as an important predictor of adverse outcomes in older surgical patients. However, evidence regarding preoperative frailty among older patients with colorectal cancer (CRC) admitted to the ICU after surgery remains limited. This study aimed to investigate the prevalence, associated factors, and impact of preoperative frailty risk on in-hospital outcomes in this high-risk population.

**Materials and methods:**

This single-center retrospective cohort study included 308 older CRC patients who underwent radical surgery and were admitted to the ICU postoperatively between June 2023 and March 2026. Preoperative frailty risk was assessed using the five-item modified Frailty Index (mFI-5). Multivariable logistic regression was used to identify factors associated with frailty risk and evaluate the association between preoperative frailty risk and postoperative complications. Generalized linear models were applied to assess the associations of frailty risk with total hospital stay, ICU stay, and hospitalization costs.

**Results:**

The prevalence of preoperative frailty risk was 55.52% in this study. Higher albumin levels were a protective factor, whereas higher blood glucose levels, elevated NLR, BMI ≥ 26.9 kg/m², polypharmacy, pain, and high fall risk were significantly associated with increased frailty risk. After adjustment for confounders, frailty risk remained an independent predictor of overall complications and severe complications and was significantly associated with prolonged total hospital stay, longer ICU stay, and increased hospitalization costs.

**Discussion:**

Preoperative frailty risk is highly prevalent among older CRC patients admitted to the ICU after surgery and is independently associated with adverse in-hospital outcomes. These findings highlight the importance of incorporating frailty assessment into perioperative management. The mFI-5 is a simple and feasible tool for preoperative risk stratification in this high-risk population and may help guide prehabilitation strategies to ultimately optimize postoperative recovery.

## 1 Introduction

Colorectal cancer (CRC) is the third most commonly diagnosed malignancy worldwide. In 2022, approximately 517,000 new cases were reported in China, accounting for 27% of global incidence (1). Although the incidence of CRC has shown a trend toward younger age in recent years, older adults remain the predominant affected population (2). Surgical resection is the mainstay of treatment for CRC. With population aging accelerating, an increasing number of older patients are undergoing radical resection (3). However, due to reduced physiological reserve and multisystem functional decline, older patients are at substantially higher surgical risk, which increases the likelihood of postoperative complications and subsequently leads to long-term functional impairment, higher mortality, and prolonged hospital stay (4). It has been reported that approximately 17.7% ~ 21.9% of patients with CRC require postoperative admission to the intensive care unit (ICU) for monitoring or organ support (5), posing additional challenges to postoperative recovery. Therefore, accurate preoperative risk stratification is essential for guiding perioperative management, optimizing resource allocation, and ultimately improving surgical outcomes.

Compared with traditional risk assessment tools such as age and the American Society of Anesthesiologists (ASA) classification, frailty has been shown to be a more robust predictor of adverse postoperative outcomes (3, 6). Frailty is a common geriatric syndrome characterized by multisystem dysregulation and reduced physiological reserve, leading to decreased tolerance to stressors and an increased risk of adverse events (7). Older CRC patients are particularly vulnerable to frailty due to tumor-related cachexia, cancer treatment, metabolic disturbances, and chronic inflammation (8). Previous studies have reported that 35.1% ~ 45.1% of older CRC patients are preoperatively frail (9, 10). Preoperative frailty has been consistently associated with increased risks of postoperative complications, mortality, and unplanned readmission (11). Notably, frailty is a reversible and preventable condition. The Society for Perioperative Assessment and Quality Improvement (SPAQI) and the American Society of Colon and Rectal Surgeons (ASCRS) both recommend incorporating frailty assessment as an essential component of preoperative evaluation in older surgical patients (12, 13). Early identification of frailty risk may facilitate timely implementation of prehabilitation strategies, thereby improving physiological reserve and postoperative recovery (6). Among various frailty assessment tools, the five-item modified Frailty Index (mFI-5) is widely used due to its simplicity and feasibility, and it has demonstrated good predictive performance for postoperative outcomes across multiple surgical populations (11).

Despite these advances, several important knowledge gaps remain. First, most existing studies have focused on cancer surgical patients treated in general wards, whereas evidence regarding frailty in postoperative cancer patients admitted to the ICU remains extremely limited. To date, only two studies have reported frailty in critically ill cancer patients and its association with short-term (14) and long-term mortality (15). Older CRC patients admitted to the ICU after surgery represent a higher-risk population with more severe clinical conditions, in whom the detrimental effects of preoperative frailty may be amplified. However, the prevalence and associated factors of preoperative frailty risk in this population remain unclear, and its association with postoperative complications has not been reported. Second, although several studies have suggested that laboratory biomarkers related to nutrition and inflammation, including albumin, neutrophil-to-lymphocyte ratio (NLR), and blood glucose, are associated with the occurrence of frailty risk in older patients (16), the findings remain inconsistent (10, 17), and these associations have not been validated in older CRC patients admitted to the ICU after surgery.

Therefore, we conducted this single-center retrospective cohort study to: (1) determine the prevalence of preoperative frailty risk among older CRC patients admitted to the ICU after surgery; (2) identify factors associated with preoperative frailty risk, with particular attention to laboratory parameters; (3) evaluate the impact of preoperative frailty risk on in-hospital outcomes, including postoperative complications, in-hospital mortality, length of hospital stay, and hospitalization costs. This study aims to provide evidence for improved perioperative risk stratification, development of targeted preoperative rehabilitation strategies, and ultimately better postoperative outcomes in this high-risk population.

## 2 Materials and methods

### 2.1 Design and participants

This single-center retrospective cohort study was conducted at a tertiary hospital in Ningxia, Yinchuan, China. This study included older CRC patients who underwent surgical resection and were admitted to the ICU postoperatively between June 2023 and March 2026. At our institution, postoperative ICU admission follows a standardized institutional process involving two pathways: planned and unplanned ICU admission. Planned ICU admission is primarily determined by colorectal surgeons based on preoperative assessment, taking into account patients’ age, major organ function, ASA classification, surgical approach, and other relevant factors. Patients considered at high risk of postoperative deterioration and potentially requiring intensive care are scheduled for direct ICU admission after surgery. Unplanned ICU admission occurs when patients require transfer to the ICU because of intraoperative events, such as hemodynamic instability, impaired oxygenation, major bleeding, surgical site infection, prolonged operative time, or other adverse events, or because of clinical deterioration after transfer to the surgical ward, including infection, bleeding, or other postoperative complications.

The inclusion criteria were as follows: (1) age ≥ 60 years, which is a commonly used age threshold for defining older adults in the Chinese healthcare system; (2) first radical resection for CRC, defined as oncologic resection of the primary tumor with adequate surgical margins and regional lymphadenectomy (18); (3) postoperative histopathological confirmation of colorectal cancer; (4) complete mFI-5 data available. The exclusion criteria were as follows: (1) emergency surgery; (2) presence of other primary malignancies; (3) preoperative receipt of neoadjuvant chemotherapy or radiotherapy; (4) preoperative acute infection, complete intestinal obstruction, intestinal perforation, or active massive hemorrhage, representing acute critical conditions. This study was approved by the Institutional Ethics Committee of the hospital (approval number: KYLL-2025-0311). This was a retrospective study, and all patient data extracted from the hospital database were de-identified and anonymized prior to analysis. Therefore, the requirement for informed consent was waived.

### 2.2 Frailty risk screening

Preoperative frailty risk was assessed using the mFI-5 developed by Subramaniam et al. (19). This index includes five components: diabetes mellitus, congestive heart failure, chronic obstructive pulmonary disease, hypertension requiring medication, and non-independent functional status. Each item is assigned one point, yielding a total score ranging from 0 to 5, with higher scores indicating greater frailty severity. Patients with a score ≥ 2 were classified as having frailty risk, whereas those with a score < 2 were classified as not having frailty risk (9). Surgeons routinely conduct a systematic admission assessment, including medical history taking, physical examination, evaluation of cardiac and pulmonary function, and monitoring of blood pressure and blood glucose levels, with the relevant information documented in the electronic medical record system. Accordingly, comorbid conditions can be determined based on the present illness history, past medical history, admission diagnoses, and relevant laboratory and examination reports documented in the electronic medical record system. Non-independent functional status was defined as requiring partial or complete assistance with activities of daily living (ADL). Before surgery, nursing staff in the surgical ward routinely assessed patients using the Barthel Index, and the assessment results were recorded in the nursing assessment form in the electronic medical record system. A Barthel Index score of 100 indicated complete independence in ADL, whereas a score < 100 indicated non-independent functional status. The mFI-5 is a simple and effective tool that has been demonstrated to effectively assess preoperative frailty risk in older CRC patients and predict clinical outcomes, and it has been recommended in the clinical practice guidelines of the American Society of Colon and Rectal Surgeons (11).

### 2.3 Demographic and clinical characteristics

We collected the following data using the institutional electronic medical record system. Demographic data included age, sex, marital status, education level, smoking history, alcohol consumption, and body mass index (BMI) (classified according to the recommended BMI range for older adults in the Chinese Dietary Guidelines (2022), including underweight ≤ 20.0 kg/m², normal weight 20.0 ~ 26.9 kg/m², and overweight/obesity ≥ 26.9 kg/m² (20)). Clinical data included prior surgical history, polypharmacy (defined as concurrent use of ≥ 5 medications), tumor differentiation, TNM stage and ICU admission type. Preoperative routinely performed risk assessment indicators included pain status (assessed using the Numerical Rating Scale), nutritional risk (assessed using the Nutritional Risk Screening 2002), fall risk (assessed using the Chinese version of the Morse Fall Scale), and VTE risk (assessed using the Caprini risk score model). Laboratory parameters were all collected from the last examination results within 3 days before surgery, including white blood cell count (WBC), lymphocyte count (LYM), neutrophil count (NEUT), serum albumin (ALB), and blood glucose (GLU), and neutrophil-to-lymphocyte ratio (NLR) was calculated. Surgical data included ASA, surgical type, operative time, and intraoperative blood transfusion. Tumor differentiation grade and TNM stage were based on postoperative pathological examination results. This study strictly adhered to institutional ethical requirements, and all collected research data were kept strictly confidential.

### 2.4 Outcomes

This study focused on in-hospital clinical outcomes after surgery, with postoperative complications as the primary outcome. The diagnosis and classification of postoperative complications were based on Chinese Expert Consensus on Standardized Diagnosis and Registration of Postoperative Complications in Gastrointestinal Tumor Surgery (2018 edition), including gastrointestinal, respiratory, and thromboembolic complications, among others (21). Severity grading was performed using the Clavien–Dindo classification system (22), with grade III or higher defined as severe complications (9). Secondary outcomes included in-hospital mortality, readmission to ICU, total length of hospital stay, length of ICU stay, and total hospitalization costs.

### 2.5 Data collection and quality control

Before data collection, the principal investigator provided standardized training to the research team on the use of the hospital electronic medical record system, the assessment procedures for the mFI-5, the classification and grading criteria for postoperative complications, and the definitions of covariates to ensure the accuracy and consistency of data collection. All data were independently reviewed and entered by two trained researchers. In the event of any discrepancies, a third senior clinician made the final determination.

### 2.6 Statistical analysis

All statistical analyses were performed using SPSS 26.0 software. For missing data, continuous variables with a normal distribution were imputed using the mean, whereas non-normally distributed continuous variables were imputed using the median; categorical variables were imputed using the mode. Continuous variables with a normal distribution were expressed as mean ± standard deviation and compared using the *t*-test. Non-normally distributed continuous variables were presented as median (interquartile range) and compared using the Mann–Whitney *U* test. Categorical variables were expressed as frequencies (percentages) and compared using the chi-square test or Fisher’s exact test. Variables with *P* < 0.05 in univariate analyses were included in multivariable logistic regression to identify independent factors associated with frailty risk. In addition, two multivariable logistic regression models were constructed with overall postoperative complications and severe complications as dependent variables, respectively, to evaluate the effect of preoperative frailty risk on postoperative outcomes. Given that total length of hospital stay, ICU stay, and total hospitalization costs were right-skewed, generalized linear models were used to analyze their association with frailty risk. All models were adjusted for potential confounding factors that may have influenced the association between frailty risk and clinical outcomes, including age, ASA, surgical type, intraoperative blood transfusion, operative time, TNM stage, and nutritional risk. To assess the robustness of the findings, multiple sensitivity analyses were performed. In addition to modeling mFI-5 as a binary variable, mFI-5 was also modeled as a continuous variable. Second, to assess whether older age influenced the association between frailty risk and clinical outcomes, the analyses were repeated after excluding patients aged ≥ 80 years. Third, to examine whether the association between frailty risk and clinical outcomes was affected by the type of ICU admission, the analyses were repeated after excluding patients with unplanned ICU admission. All sensitivity analyses were adjusted for the same covariates as the original binary mFI-5 models. All statistical tests were two-sided, and *P* < 0.05 was considered statistically significant.

## 3 Results

### 3.1 Baseline characteristics of patients

During the study period, approximately 2,013 patients underwent radical resection for colorectal cancer at our institution, of whom approximately 1,233 were aged ≥ 60 years. Among all patients who underwent radical resection for colorectal cancer, 396 (approximately 19.67%) were admitted to the ICU postoperatively. Among these ICU-admitted patients, 308 met the inclusion and exclusion criteria and were included in the final analysis. Missing data accounted for < 3% and were imputed using mode imputation. The median age of the cohort was 74.50 years (range, 60~89 years), with 88 (28.57%) aged 60~69 years, 145 (47.08%) aged 70~79 years, and 75 (24.35%) aged ≥ 80 years. The cohort was predominantly male (62.66%). More than half of the patients (51.95%) had a primary school education or lower. Most patients were classified as ASA grade III (78.90%), and the vast majority underwent laparoscopic surgery (94.48%). Regarding ICU admission type, 281 patients (91.23%) had planned ICU admission, while 27 (8.77%) had unplanned ICU admission. According to the mFI-5 classification, 171 patients (55.52%) were identified as having preoperative frailty risk (mFI-5 ≥ 2), while 137 patients (44.48%) were classified as not having frailty risk (mFI-5 < 2). The comparison of baseline characteristics between the two groups is presented in **Table 1**.

**Table 1:** Characteristics of older colorectal cancer patients requiring ICU admission after surgery.

Characteristic	All patients	No frailty risk	Frailty risk	Test statistic	*P* value
	(*n* = 308)	mFI-5 < 2 (*n* = 137)	mFI-5 ≥ 2 (*n* = 171)		
Age [years, *M*(*P*_25_, *P*_75_)]	74.50 (68.00, 79.00)	73.00 (67.00, 79.00)	75.00 (70.00, 80.00)	-2.686^3)^	0.007
Sex, *n* (%)				2.127^2)^	0.145
Male	193 (62.66)	92 (67.15)	101 (59.06)		
Female	115 (37.34)	45 (32.85)	70 (40.94)		
Marital status, *n* (%)				0.040^2)^	0.841
Married	296 (96.10)	132 (96.35)	164 (95.91)		
Widowed/Divorced/ Unmarried	12 (3.90)	5 (3.65)	7 (4.09)		
Education level, *n* (%)				2.211^2)^	0.331
Primary school or below	160 (51.95)	69 (50.36)	91 (53.22)		
Junior high school	81 (26.30)	33 (24.09)	48 (28.07)		
Senior high school or above	67 (21.75)	35 (25.55)	32 (18.71)		
Smoking history, *n* (%)				1.536^2)^	0.215
No	233 (75.65)	99 (72.26)	134 (78.36)		
Yes	75 (24.35)	38 (27.74)	37 (21.64)		
Alcohol consumption, *n* (%)				0.221^2)^	0.638
No	262 (85.06)	118 (86.13)	144 (84.21)		
Yes	46 (14.94)	19 (13.87)	27 (15.79)		
BMI, *n* (%)				21.829^2)^	< 0.001
≤ 20.0	53 (17.21)	14 (10.22)	39 (22.81)		
20.0 ~ 26.9	199 (64.61)	108 (78.83)	91 (53.22)		
≥ 26.9	56 (18.18)	15 (10.95)	41 (23.98)		
Prior surgical history, *n* (%)				9.240^2)^	0.002
No	128 (41.56)	70 (51.09)	58 (33.92)		
Yes	180 (58.44)	67 (48.91)	113 (66.08)		
Polypharmacy, *n* (%)				19.963^2)^	< 0.001
No	267 (86.69)	132 (96.35)	135 (78.95)		
Yes	41 (13.31)	5 (3.65)	36 (21.05)		
Tumor differentiation, *n* (%)				4.136^2)^	0.126
Poorly differentiated	22 (7.14)	6 (4.38)	16 (9.36)		
Moderately differentiated	161 (52.27)	69 (50.36)	92 (53.80)		
Well differentiated	125 (40.58)	62 (45.26)	63 (36.84)		
TNM stage, *n* (%)				2.075^2)^	0.557
0/I	49 (15.91)	26 (18.98)	23 (13.45)		
II	135 (43.83)	60 (43.80)	75 (43.86)		
III	96 (31.17)	40 (29.20)	56 (32.75)		
IV	28 (9.09)	11 (8.03)	17 (9.94)		
Pain status, *n* (%)				26.675^2)^	< 0.001
No	177 (57.47)	101 (73.72)	76 (44.44)		
Yes	131 (42.53)	36 (26.28)	95 (55.56)		
Nutritional risk, *n* (%)				1.748^2)^	0.186
No	120 (38.96)	59 (43.07)	61 (35.67)		
Yes	188 (61.04)	78 (56.93)	110 (64.33)		
Fall risk, *n* (%)				90.272^2)^	< 0.001
Low	57 (18.51)	50 (36.50)	7 (4.09)		
Moderate	53 (17.21)	38 (27.74)	15 (8.77)		
High	198 (64.29)	49 (35.77)	149 (87.13)		
VTE risk, *n* (%)				12.961^2)^	0.002
Low	33 (10.71)	22 (16.06)	11 (6.43)		
Moderate	86 (27.92)	45 (32.85)	41 (23.98)		
High	189 (61.36)	70 (51.09)	119 (69.59)		
WBC [×10^9^/L, *M*(*P*_25_, *P*_75_)]	6.16 (4.84, 7.28)	6.02 (4.58, 7.16)	6.22 (4.95, 7.32)	-0.814^3)^	0.416
LYM [×10^9^/L, *M*(*P*_25_, *P*_75_)]	1.44 (1.07, 1.81)	1.53 (1.17, 1.98)	1.29 (0.97, 1.66)	-3.536^3)^	< 0.001
NEUT [×10^9^/L, *M*(*P*_25_, *P*_75_)]	3.89 (2.88, 5.34)	3.56 (2.46, 4.90)	4.36 (3.11, 5.81)	-3.201^3)^	0.001
NLR [*M*(*P*_25_, *P*_75_)]	2.70 (1.85, 3.99)	2.28 (1.58, 3.35)	2.98 (2.05, 5.25)	-4.198^3)^	< 0.001
ALB (g/L,*x̄ ± s*)	36.39 ± 4.88	38.04 ± 4.75	35.07 ± 4.59	5.543^1)^	< 0.001
GLU [mmol/L, *M*(*P*_25_, *P*_75_)]	5.32 (4.77, 6.62)	5.18 (4.61, 5.60)	5.52 (4.85, 7.21)	-5.029^3)^	< 0.001
ASA, *n* (%)				12.386^2)^	0.002
II	50 (16.23)	31 (22.63)	19 (11.11)		
III	243 (78.90)	104 (75.91)	139 (81.29)		
IV	15 (4.87)	2 (1.46)	13 (7.60)		
Surgery type, *n* (%)				0.048^2)^	0.826
Laparoscopic	291 (94.48)	129 (94.16)	162 (94.74)		
Open	17 (5.52)	8 (5.84)	9 (5.26)		
Intraoperative blood transfusion, *n* (%)				0.011^2)^	0.917
No	251 (81.49)	112 (81.75)	139 (81.29)		
Yes	57 (18.51)	25 (18.25)	32 (18.71)		
Operative time [h, *M*(*P*_25_, *P*_75_)]	3.50 (3.00, 4.25)	3.50 (3.00, 4.22)	3.50 (3.00, 4.31)	-0.696^3)^	0.487
ICU admission type, *n* (%)				4.126^2)^	0.042
Planned admission	281 (91.23)	130 (94.89)	151 (88.30)		
Unplanned admission	27 (8.77)	7 (5.11)	20 (11.70)		

1) *t* value; 2) *χ²* value; 3) *Z* value.

### 3.2 Distribution of mFI-5 components and scores

As shown in **Table 2**, the most common mFI-5 component was non-independent functional status, which was present in 79.87% of the overall cohort, followed by hypertension requiring medication (53.25%) and diabetes mellitus (20.78%). Regarding the distribution of mFI-5 scores, 104 patients (75.91%) in the no frailty risk group had an mFI-5 score of 1. In contrast, patients with frailty risk were predominantly distributed in scores of 2 ~ 3 (91.23%), with only a small proportion classified as scores 4 ~ 5.

**Table 2:** Distribution of mFI-5 scores and component items.

Variable	All patients (*n* = 308)	No frailty risk (*n* = 137)	Frailty risk (*n* = 171)
mFI-5 components, *n* (%)			
Non-independent functional status	246 (79.87)	83 (60.58)	163 (95.32)
Hypertension requiring medication	164 (53.25)	13 (9.49)	151 (88.30)
Diabetes mellitus	64 (20.78)	6 (4.38)	58 (33.92)
Chronic obstructive pulmonary disease	31 (10.06)	1 (0.73)	30 (17.54)
Congestive heart failure	22 (7.14)	1 (0.73)	21 (12.28)
mFI-5 score distribution, *n* (%)			
0	33 (10.71)	33 (24.09)	0
1	104 (33.77)	104 (75.91)	0
2	106 (34.42)	0	106 (61.99)
3	50 (16.23)	0	50 (29.24)
4	14 (4.55)	0	14 (8.19)
5	1 (0.32)	0	1 (0.58)

### 3.3 Independent factors associated with preoperative frailty risk

Multivariable logistic regression analysis identified several independent factors associated with preoperative frailty risk, as shown in **Table 3**. Higher ALB was a protective factor for frailty risk (*OR* = 0.896). In contrast, higher GLU (*OR* = 1.476) and higher NLR (*OR* = 1.139) were independently associated with an increased risk of frailty. Compared with patients with normal BMI (20.0~26.9 kg/m²), those with BMI ≥ 26.9 kg/m² had a significantly higher risk of frailty (*OR* = 3.948). Additionally, polypharmacy (*OR* = 5.854), presence of pain (*OR* = 3.082), and high fall risk (*OR* = 6.976) were significantly associated with frailty risk.

**Table 3:** Multivariable logistic regression analysis of factors associated with preoperative frailty risk.

Characteristic	*B*	*SE*	*Wald χ^2^*	*OR* (95% *CI*)	*P* value
ALB	-0.109	0.034	10.286	0.896 (0.839, 0.958)	**0.001**
GLU	0.389	0.112	12.133	1.476 (1.186, 1.837)	**< 0.001**
NLR	0.130	0.063	4.300	1.139 (1.007, 1.288)	**0.038**
BMI (reference: 20.0 ~ 26.9)			10.084		0.006
≤ 20.0	0.628	0.454	1.915	1.874 (0.770, 4.559)	0.166
≥ 26.9	1.373	0.457	9.039	3.948 (1.613, 9.663)	**0.003**
Polypharmacy	1.767	0.591	8.947	5.854 (1.839, 18.636)	**0.003**
Pain status	1.126	0.338	11.072	3.082 (1.588, 5.981)	**< 0.001**
Fall risk (reference: Low)			21.147		< 0.001
Moderate	0.885	0.491	3.241	2.422 (0.925, 6.347)	0.072
High	1.943	0.451	18.540	6.976 (2.881, 16.891)	**< 0.001**
Age	0.036	0.025	2.194	1.037 (0.988, 1.088)	0.139
VTE risk (reference: Low)			2.035		0.362
Moderate	0.528	0.556	0.903	1.696 (0.571, 5.038)	0.342
High	0.751	0.535	1.970	2.119 (0.742, 6.049)	0.160
Prior surgical history	0.441	0.315	1.953	1.554 (0.837, 2.884)	0.162

Bold values indicate statistically significant results (*P* < 0.05).

### 3.4 In-hospital outcomes in patients with and without frailty risk

The comparison of in-hospital outcomes between patients with and without frailty risk is shown in **Table 4**. The overall complication rate, as well as the incidence of Clavien–Dindo grade ≥ III and grade ≥ IV complications, was significantly higher in the frailty risk group compared with the no frailty risk group. Regarding specific types of complications, patients with frailty risk had significantly higher rates of gastrointestinal, respiratory, thromboembolic, and infectious complications than non-frail patients. In the frailty risk group, 3 patients experienced in-hospital mortality and 4 patients required ICU readmission, whereas no in-hospital mortality and 1 ICU readmission were observed in the no frailty risk group. However, no statistically significant differences were observed between the two groups. In addition, patients with frailty risk had longer total hospital stay and ICU stay, as well as higher hospitalization costs compared with those without frailty risk.

**Table 4:** Comparison of in-hospital outcomes between patients with and without frailty risk.

Outcome	All patients (*n* = 308)	No frailty risk (*n* = 137)	Frailty risk (*n* = 171)	*P* value
Overall complications, *n* (%)	150 (48.70)	46 (33.58)	104 (60.82)	**< 0.001**
Clavien-Dindo grade ≥ III, *n* (%)	69 (22.40)	18 (13.14)	51 (29.82)	**< 0.001**
Clavien-Dindo grade ≥ IV, *n* (%)	19 (6.17)	4 (2.92)	15 (8.77)	**0.034**
Gastrointestinal complications, *n* (%)	32 (10.39)	8 (5.84)	24 (14.04)	**0.019**
Respiratory complications, *n* (%)	93 (30.19)	23 (16.79)	70 (40.94)	**< 0.001**
Cardiovascular complications, *n* (%)	15 (4.87)	3 (2.19)	12 (7.02)	0.050
Thromboembolic complications, *n* (%)	36 (11.69)	10 (7.30)	26 (15.20)	**0.032**
Urinary system complications, *n* (%)	4 (1.30)	1 (0.73)	3 (1.75)	0.777*
Infectious complications, *n* (%)	99 (32.14)	35 (25.55)	64 (37.43)	**0.027**
In-hospital mortality, *n* (%)	3 (0.97)	0	3 (1.75)	0.330*
Readmission to ICU, *n* (%)	5 (1.62)	1 (0.73)	4 (2.34)	0.511*
Total hospital length of stay [d, *M*(*P*_25_, *P*_75_)]	17.00 (14.00, 21.00)	16.00 (14.00, 21.00)	18.00 (15.00, 22.00)	**0.004**
ICU length of stay [d, *M*(*P*_25_, *P*_75_)]	1.00 (1.00, 2.00)	1.00 (1.00, 2.00)	1.00 (1.00, 2.00)	**0.025**
Total hospitalization cost [10,000 RMB, *M*(*P*_25_, *P*_75_)]	10.90 (7.99, 13.48)	9.94 (7.58, 12.49)	11.82 (8.68, 13.90)	**0.002**

*P values were calculated using the continuity-corrected chi-square test.

Bold values indicate statistically significant results (*P* < 0.05).

### 3.5 Association between frailty risk and in-hospital outcomes

Multivariable logistic regression models were constructed with overall postoperative complications and severe complications (Clavien–Dindo grade ≥ III) as dependent variables, adjusting for age, ASA, surgical type, intraoperative blood transfusion, operative time, TNM stage, and nutritional risk. The results of the regression analysis are shown in **Table 5**. Preoperative frailty risk was significantly associated with an increased risk of overall complications (*OR* = 3.016, 95% *CI*: 1.821 ~ 4.998, *P* < 0.001) and Clavien–Dindo grade ≥ III complications (*OR* = 2.627, 95% *CI*: 1.385 ~ 4.983, *P* = 0.003). Generalized linear model analyses are shown in **Table 6** and **Supplementary Table S1**. After adjusting for the above confounding factors, preoperative frailty risk remained significantly associated with prolonged total hospital stay (Exp(B) = 1.184, 95% *CI*: 1.096 ~ 1.278, *P* < 0.001), extended ICU stay (Exp(B) = 1.323, 95% *CI*: 1.127 ~ 1.554, *P* < 0.001), and increased total hospitalization costs (Exp(B) = 1.115, 95% *CI*: 1.019 ~ 1.220, *P* = 0.018).

**Table 5:** Multivariable logistic regression analysis of postoperative complications.

Variable	Overall complications		Severe complications (Clavien-Dindo grade ≥ III)	
	*OR* (95% *CI*)	*P* value	*OR* (95% *CI*)	*P* value
Frailty risk	3.016 (1.821, 4.998)	**< 0.001**	2.627 (1.385, 4.983)	**0.003**
Age	1.002 (0.967, 1.039)	0.903	1.046 (1.000, 1.093)	0.048
ASA (reference: II)		0.290		0.332
III	1.274 (0.641, 2.531)	0.490	0.762 (0.333, 1.743)	0.520
IV	3.175 (0.752, 13.401)	0.116	1.766 (0.428, 7.283)	0.431
Surgery type	0.873 (0.284, 2.686)	0.813	2.834 (0.885, 9.075)	0.079
Intraoperative blood transfusion	1.912 (0.978, 3.737)	0.058	1.990 (0.986, 4.016)	0.055
Operative time	1.449 (1.155, 1.818)	0.001	1.169 (0.921, 1.485)	0.199
TNM stage (reference: 0/I)		0.390		0.821
II	0.654 (0.318, 1.344)	0.248	1.095 (0.458, 2.619)	0.838
III	1.031 (0.482, 2.207)	0.937	0.867 (0.338, 2.223)	0.767
IV	0.985 (0.350, 2.775)	0.978	0.687 (0.189, 2.500)	0.569
Nutritional risk	1.553 (0.925, 2.609)	0.096	1.221 (0.659, 2.264)	0.525

Bold values indicate statistically significant results (*P* < 0.05).

**Table 6:** Generalized linear model analysis of total hospital length of stay, ICU length of stay, and total hospitalization cost.

Outcome	Exp(B) (95% *CI*)	*P* value
Total hospital length of stay	1.184 (1.096, 1.278)	**< 0.001**
ICU length of stay	1.323 (1.127, 1.554)	**< 0.001**
Total hospitalization cost	1.115 (1.019, 1.220)	**0.018**

All models were adjusted for age, ASA classification, surgical type, intraoperative blood transfusion, operative time, TNM stage, and nutritional risk. The complete model output, including all covariates, is provided in **Supplementary Table S1**.

Bold values indicate statistically significant results (*P* < 0.05).

### 3.6 Sensitivity analyses

Three sensitivity analyses were performed. First, mFI-5 was modeled as a continuous variable. Each 1-point increase in the mFI-5 score was significantly associated with overall complications (*OR* = 1.605, 95% *CI*: 1.243 ~ 2.074, *P* < 0.001), severe complications (*OR* = 1.494, 95% *CI*: 1.115 ~ 2.001, *P* = 0.007), total hospital length of stay (Exp(B) = 1.083, 95% *CI*: 1.043 ~ 1.124, *P* < 0.001), and ICU stay (Exp(B) = 1.189, 95% *CI*: 1.105 ~ 1.279, *P* < 0.001), but not hospitalization cost (Exp(B) = 1.035, 95% *CI*: 0.991 ~ 1.081, *P* = 0.125) (**Supplementary Tables S2**, **S3**). Second, after excluding 75 patients aged ≥ 80 years, frailty risk remained significantly associated with overall complications (*OR* = 2.986, 95% *CI*: 1.638 ~ 5.443, *P* < 0.001), severe complications (*OR* = 2.753, 95% *CI*: 1.208 ~ 6.276, *P* = 0.016), total hospital length of stay (Exp(B) = 1.249, 95% *CI*: 1.139 ~ 1.369, *P* < 0.001), and hospitalization cost (Exp(B) = 1.146, 95% *CI*: 1.031 ~ 1.274, *P* = 0.012), but not ICU stay (Exp(B) = 1.197, 95% *CI*: 0.989 ~ 1.448, *P* = 0.065) (**Supplementary Tables S4**, **S5**). Third, after excluding 27 patients with unplanned ICU admission, frailty risk remained significantly associated with overall complications (*OR* = 3.053, 95% *CI*: 1.788~5.213, *P* < 0.001), severe complications (*OR* = 2.665, 95% *CI*: 1.303 ~ 5.454, *P* = 0.007), total hospital length of stay (Exp(B) = 1.141, 95% *CI*: 1.058 ~ 1.231, *P* < 0.001), ICU stay (Exp(B) = 1.131, 95% *CI*: 1.022 ~ 1.252, *P* = 0.017), and hospitalization cost (Exp(B) = 1.154, 95% *CI*: 1.064 ~ 1.251, *P* < 0.001) (**Supplementary Tables S6**, **S7**).

## 4 Discussion

To our knowledge, this is the first study to investigate preoperative frailty risk in older CRC patients who were admitted to the ICU after surgery. We found that the prevalence of preoperative frailty risk in this population was as high as 55.52% and identified several clinical and laboratory factors independently associated with frailty risk. Furthermore, preoperative frailty risk was independently associated with multiple adverse in-hospital outcomes, including postoperative complications and prolonged hospital length of stay. These findings highlight the importance of incorporating frailty assessment into perioperative management for this high-risk population, which may facilitate early risk stratification, enable the development of individualized prehabilitation strategies, and optimize postoperative recovery and healthcare resource allocation.

The prevalence of preoperative frailty risk in our study was significantly higher than that reported in previous studies involving general older CRC patients (9, 10), which may be related to the fact that all patients in our study required postoperative ICU admission. In our institution, patients identified as high risk during preoperative assessment are generally planned for postoperative ICU admission, which may have resulted in a study population with more severe baseline conditions, higher surgical risks, or greater perioperative physiological instability. Furthermore, the prevalence of frailty risk observed in our study was also substantially higher than the 25.2% reported by Osatnik among critically ill patients with cancer (14). This difference may be partly explained by the older age of our study population, with a median age of 74.50 years, whereas their study included adult patients with cancer aged ≥ 18 years. Furthermore, the frailty assessment tools and timing of assessment differed between the two studies. They used the Clinical Frailty Scale (CFS) and assessed frailty risk at ICU admission, whereas frailty risk in our study was evaluated preoperatively using the mFI-5. Notably, we also found that 75.91% of patients in the no frailty risk group had an mFI-5 score of 1 point, indicating that they were already in a prefrailty state. These findings suggest that frailty risk is highly prevalent among older CRC patients requiring postoperative ICU admission and warrants greater clinical attention.

Our study also identified several factors associated with preoperative frailty risk, among which laboratory indicators reflecting nutritional status, metabolism, and inflammation were particularly noteworthy. Higher ALB were identified as a protective factor (*OR* = 0.896), whereas higher GLU (*OR* = 1.476) and higher NLR (*OR* = 1.139) were independent risk factors for frailty risk. Albumin reflects nutritional reserves and inflammatory status in the body (23). Older CRC patients are prone to malnutrition before surgery due to reduced appetite and limited physical activity. Meanwhile, cancer-related chronic inflammation may increase capillary permeability and accelerate albumin loss, thereby affecting muscle quality and physical function and increasing the risk of frailty (24). These findings suggest that clinicians should pay attention to patients with lower preoperative albumin levels and consider individualized nutritional support strategies in collaboration with nutrition specialists when necessary. Persistent hyperglycemia may induce oxidative stress and inflammatory responses, accelerate muscle protein breakdown, and inhibit protein synthesis (25). Therefore, dynamic glucose monitoring and management are recommended for patients with elevated preoperative blood glucose levels or concomitant diabetes. As a novel inflammatory marker, NLR reflects systemic inflammation and immune dysregulation and has been closely associated with the development of frailty (16). Previous studies have shown that both excessively high and low BMI are associated with an increased risk of frailty in older adults (26). In our study, BMI ≥ 26.9 (*OR* = 3.948) was identified as an independent risk factor for frailty risk among older CRC patients. This association may be related to adipose accumulation-induced production of pro-inflammatory cytokines, which may impair cardiovascular, immune, and muscular system functions (26). Our study also found that pain (*OR* = 3.082) was independently associated with preoperative frailty risk. Persistent pain may contribute to frailty by impairing mobility, reducing resting metabolic rate, decreasing nutritional intake, and compromising sleep quality (27). This highlights the importance of preoperative pain assessment and the implementation of stepwise pain management strategies. Furthermore, polypharmacy (*OR* = 5.854) and high fall risk (*OR* = 6.976) were also associated with frailty risk. Multimorbidity is a common condition among older adults and has a bidirectional relationship with frailty (28). Multimorbidity is often accompanied by polypharmacy, while adverse drug interactions and reduced drug metabolism capacity in older adults may further increase the risk of frailty (29). Therefore, clinicians should assess comorbidities before surgery, comprehensively consider treatment strategies, and simplify medication regimens whenever possible. Patients with a high risk of falls often have gait instability, impaired sensory function, and balance disturbances, and may reduce physical activity due to fear of falling, which may subsequently contribute to declines in muscle strength and bone density (30). Clinicians should consider interventions such as balance training, fall prevention education, and encouraging family-assisted activities to enhance patients’ confidence in physical activity, improve adherence to preoperative activity, and prevent or alleviate preoperative frailty. However, these associations should be interpreted cautiously because polypharmacy and high fall risk may potentially overlap with the comorbidity and non-independent functional status components of the mFI-5.

Furthermore, older CRC patients with frailty risk admitted to the ICU after surgery had poorer in-hospital outcomes than those without frailty risk. The frailty risk group showed significantly higher rates of overall complications, severe complications, and gastrointestinal, respiratory, thromboembolic, and infectious complications. After adjustment for age, ASA, operative time, and other confounders, frailty risk remained an independent predictor of overall complications (*OR* = 3.016) and severe complications (*OR* = 2.627). Notably, age was independently associated with severe complications (*OR* = 1.046, *P* = 0.048) but not overall complications (*P* = 0.903), whereas ASA was not associated with either outcome. These findings suggest that chronological age and ASA alone may have limited value in predicting postoperative complications. In contrast, frailty risk screening provides more consistent predictive value for both overall and severe complications by capturing multidimensional functional deficits. Our findings are consistent with Huang, who reported that mFI-5 ≥ 2 independently predicted severe complications (*OR* = 2.254) in 208 older CRC patients undergoing surgery in Guangdong, China (9). However, Ogata found no association between mFI-5 and 30-day severe complications in 299 older Japanese CRC patients (10). This heterogeneity may be explained by differences in study populations, perioperative management, and complication definitions. Despite these inconsistencies, the clinical value of preoperative frailty risk assessment in this population remains important. Although in-hospital mortality, ICU readmission, and cardiovascular, and urinary complications were numerically higher in the frailty risk group, these differences were not statistically significant, possibly due to the low event rates and limited statistical power. Our study further found that frailty risk independently prolonged total hospital stay, ICU stay and increased hospitalization costs. This finding is consistent with Ling (15) and highlights the substantial economic burden of preoperative frailty risk in this population. Importantly, when mFI-5 was modeled as a continuous variable, the results suggested that the risk of adverse outcomes may increase with increasing mFI-5 scores. Although the association between frailty risk and hospitalization costs was no longer statistically significant in this analysis, this finding may be partly attributable to the substantial variability in hospitalization costs and the limited number of patients with higher mFI-5 scores and should therefore be interpreted cautiously. Furthermore, the main findings remained generally consistent after excluding older patients and those with unplanned ICU admission, which may provide some support for the robustness of the associations between preoperative frailty risk and adverse in-hospital outcomes across different age groups and ICU admission types. However, the relatively small sample size limited our ability to conduct age-stratified analyses and further classify frailty into more granular levels. Future large-scale, multicenter studies are warranted to further investigate these associations across different age groups and levels of frailty severity.

Our findings have several important clinical implications. First, the high prevalence of frailty risk among older CRC patients admitted to the ICU highlights the urgent need for routine preoperative frailty screening. However, frailty screening remains underused in current surgical practice. A survey by the International Society of Geriatric Oncology among surgical oncologists in the United States and Europe showed that although 90% of respondents routinely used preoperative tools to assess surgical risk (most commonly ASA, physical performance, and nutritional status), fewer than one-quarter used frailty assessment tools (31). Comprehensive geriatric assessment (CGA) is regarded as the gold standard for identifying frailty in older adults; however, its implementation is limited by the time and resources required (32). In contrast, mFI-5 can be rapidly derived from routine medical history and functional assessments and has good predictive performance, making it more feasible for large-scale clinical implementation (33). Second, currently used frailty assessment tools mainly rely on clinical indicators, including comorbidities, functional status, and physical performance, which may not fully capture early or potential changes associated with frailty (16). Given the close association between frailty and chronic low-grade inflammation as well as nutritional-metabolic disturbances in older adults, laboratory indicators such as albumin, glucose, and NLR may provide additional value for the early identification and assessment of frailty. Importantly, the value of incorporating mFI-5 into preoperative assessment lies not only in risk identification but also in guiding risk management. This not only provides an opportunity to optimize patients’ physiological reserve and improve their outcomes, but also provides useful information to support appropriate perioperative ICU utilization and allocation of medical resources. This concept is supported by Hall, who evaluated a preoperative frailty screening initiative involving 9,153 surgical patients (34). Patients identified as frail were communicated to surgical, anesthesiology, and critical care teams, and perioperative care plans were adjusted based on multidisciplinary input. The study found that the implementation of the frailty screening initiative was associated with lower 30-day, 6-month, and 1-year mortality, with more pronounced benefits observed among frail patients. Currently, multimodal prehabilitation incorporating exercise, nutrition, and psychological support has emerged as a promising strategy to improve frailty and postoperative outcomes (13). The factors identified in our study, including polypharmacy, pain, and high fall risk, also represent potential targets for prehabilitation interventions.

This study has several limitations. First, the single-center retrospective design may limit the generalizability of our findings. In particular, our institution admits patients with colorectal cancer to the ICU postoperatively when they are identified as being at high risk based on preoperative assessment, resulting in a relatively high ICU admission rate. Given that ICU admission criteria vary across institutions, our findings may not be directly generalizable to medical centers with different ICU admission practices. Future multicenter studies involving institutions with different ICU admission practices are warranted to validate our findings. Second, although we adjusted for potential confounders, the influence of unmeasured variables cannot be completely excluded, such as R0 resection status, lymph node yield, and treatments and medications administered after ICU admission. Third, mFI-5 does not capture important dimensions such as cognitive and nutritional status and should therefore be regarded primarily as a frailty risk-screening tool. Moreover, functional status was retrospectively assessed using the Barthel Index, which may have overestimated the degree of functional dependence. Fourth, the relatively small sample size limited the statistical power for age-stratified analyses, comparisons across different frailty score levels, and detecting differences in some secondary outcomes. Finally, outcome data were limited to the in-hospital period, without follow-up of post-discharge functional recovery and long-term health outcomes, which are important for fully understanding the long-term impact of frailty. Despite these limitations, our study provides valuable clinical insights into the growing high-risk population of older CRC patients requiring postoperative ICU admission and emphasizes the need for frailty assessment and intervention. Future multicenter prospective studies with larger samples are warranted to validate our findings and further evaluate whether prehabilitation interventions can improve postoperative outcomes in this vulnerable population.

## 5 Conclusion

Preoperative frailty risk is common among older CRC patients admitted to the ICU after surgery and is independently associated with adverse in-hospital outcomes. The mFI-5 may serve as an effective tool for preoperative risk stratification in this population, while routine laboratory indicators may provide complementary information for frailty screening. Incorporating frailty assessment into perioperative management may facilitate early identification of high-risk patients and guide individualized prehabilitation strategies.

## Data Availability

The raw data supporting the conclusions of this article will be made available by the authors, without undue reservation.

## References

[1] Wu S Zhang Y Lin Z Wei M . Global burden of colorectal cancer in 2022 and projections to 2050: incidence and mortality estimates from GLOBOCAN. BMC Cancer. (2025) 25:1770. doi: 10.1186/s12885-025-15138-0 41239247 PMC12619147

[2] Siegel RL Wagle NS Cercek A Smith RA Jemal A . Colorectal cancer statistics, 2023. CA Cancer J Clin. (2023) 73:233–54. doi: 10.3322/caac.21772 36856579

[3] Taniguchi H Kumamoto T Otsuka K Cheong Y Omura Y Hiro J . Prognostic value of the Clinical Frailty Scale for outcomes after minimally invasive rectal cancer surgery in older patients. J Surg Oncol. (2026) 133:743–53. doi: 10.1002/jso.70227 41787823

[4] Xiang M Luo A Wang Y Lin J Xiong Z Hou F . Analysis of risk factors for elective surgery complications in a cancer specialty hospital based on propensity score matching. BMC Health Serv Res. (2025) 25:834. doi: 10.1186/s12913-025-13065-1 40598283 PMC12210737

[5] Wang L Wu Y Deng L Tian X Ma J . Construction and validation of a risk prediction model for postoperative ICU admission in patients with colorectal cancer: clinical prediction model study. BMC Anesthesiol. (2024) 24:222. doi: 10.1186/s12871-024-02598-3 38965472 PMC11223334

[6] Ethun CG Bilen MA Jani AB Maithel SK Ogan K Master VA . Frailty and cancer: Implications for oncology surgery, medical oncology, and radiation oncology. CA Cancer J Clin. (2017) 67:362–77. doi: 10.3322/caac.21406 28731537

[7] Canter BE Evans EA . Frailty in older adults. N Engl J Med. (2024) 391:1758–9. doi: 10.1056/NEJMc2411327 39504532

[8] Zhong M Wu X Li X Xie J Wang X Du Q . A nomogram model for predicting frailty in middle-aged and elderly patients with colorectal cancer: a cross-sectional study. Eur J Oncol Nurs. (2024) 73:102718. doi: 10.1016/j.ejon.2024.102718 39522257

[9] Huang L Li Z Jian M Wu X Chen H Qin H . Application of MFI-5 in severe complications and unfavorable outcomes after radical resection of colorectal cancer. World J Surg Oncol. (2023) 21:307. doi: 10.1186/s12957-023-03186-4 37752577 PMC10521557

[10] Ogata T Sadakari Y Nakane H Koikawa K Kanno H Kohata R . The five-item modified frailty index predicts long-term outcomes in elderly patients undergoing colorectal cancer surgery. World J Surg Oncol. (2023) 21:268. doi: 10.1186/s12957-023-03150-2 37626381 PMC10463643

[11] Xia L Yin R Mao L Shi X . Prevalence and impact of frailty in patients undergoing colorectal cancer surgery: a systematic review and meta-analysis based on modified frailty index. J Surg Oncol. (2024) 130:604–12. doi: 10.1002/jso.27778 39016206

[12] Alvarez-Nebreda ML Bentov N Urman RD Setia S Huang JC Pfeifer K . Recommendations for preoperative management of frailty from the Society for Perioperative Assessment and Quality Improvement (SPAQI). J Clin Anesth. (2018) 47:33–42. doi: 10.1016/j.jclinane.2018.02.011 29550619

[13] Saur NM Davis BR Montroni I Shahrokni A Rostoft S Russell MM . The american society of colon and rectal surgeons clinical practice guidelines for the perioperative evaluation and management of frailty among older adults undergoing colorectal surgery. Dis Colon Rectum. (2022) 65:473–88. doi: 10.1097/DCR.0000000000002410 35001046

[14] Osatnik J Matarrese A Leone B Cesar G Kleinert M Sosa F . Frailty and clinical outcomes in critically ill patients with cancer: a cohort study. J Geriatr Oncol. (2022) 13:1156–61. doi: 10.1016/j.jgo.2022.08.006 36031524

[15] Ling RR Ueno R Alamgeer M Sundararajan K Sundar R Bailey M . Frailty in Australian patients admitted to Intensive care unit after elective cancer-related surgery: a retrospective multicentre cohort study (FRAIL-CANCER-SURG study). Br J Anaesth. (2024) 132:695–706. doi: 10.1016/j.bja.2024.01.020 38378383

[16] Zeng M Li Y Zhu Y Sun Y . Inflammatory markers and clinical factors as key independent risk factors for frailty: a retrospective study. BMC Geriatr. (2025) 25:404. doi: 10.1186/s12877-025-06033-1 40468183 PMC12135400

[17] Cadwell JB Afonso AM Shahrokni A . Prognostic nutritional index (PNI), independent of frailty is associated with six-month postoperative mortality. J Geriatr Oncol. (2020) 11:880–4. doi: 10.1016/j.jgo.2020.03.013 32253157 PMC8311543

[18] Medical Administration Department, National Health Commission of the People’s Republic of China Chinese Society of Oncology . National Health Commission of China: colorectal cancer diagnosis and treatment guidelines (2025 edition) (concise version). Chin J Surg. (2026) 64:89–95. doi: 10.3760/cma.j.cn112139-20251205-00577 41667920

[19] Subramaniam S Aalberg JJ Soriano RP Divino CM . New 5-factor modified frailty index using american college of surgeons NSQIP data. J Am Coll Surg. (2018) 226:173–181.e8. doi: 10.1016/j.jamcollsurg.2017.11.005 29155268

[20] Chinese Nutrition Society . Dietary Guidelines for Older Adults in China (2022). Beijing: People's Medical Publishing House (2022).

[21] Li ZY Wu ZQ Ji JF . Chinese expert consensus on standardized diagnosis and registration of postoperative complications in gastrointestinal tumor surgery (2018 edition). Chin J Pract Surg. (2018) 38:589–95. doi: 10.19538/j.cjps.issn1005-2208.2018.06.01

[22] Katayama H Kurokawa Y Nakamura K Ito H Kanemitsu Y Masuda N . Extended Clavien-Dindo classification of surgical complications: Japan Clinical Oncology Group postoperative complications criteria. Surg Today. (2016) 46:668–85. doi: 10.1007/s00595-015-1236-x 26289837 PMC4848327

[23] Allison SP Lobo DN . The clinical significance of hypoalbuminaemia. Clin Nutr. (2024) 43:909–14. doi: 10.1016/j.clnu.2024.02.018 38394971

[24] Di Meglio A Vaz-Luis I . Systemic inflammation and cancer-related frailty: shifting the paradigm toward precision survivorship medicine. ESMO Open. (2024) 9:102205. doi: 10.1016/j.esmoop.2023.102205 38194879 PMC10820355

[25] Lisco G Disoteo OE De Tullio A De Geronimo V Giagulli VA Monzani F . Sarcopenia and diabetes: a detrimental liaison of advancing age. Nutrients. (2023) 16:63. doi: 10.3390/nu16010063 38201893 PMC10780932

[26] Zeng P Jiang C Yin H Zhou M He H Yin D . Weight-adjusted waist index and deficit accumulation frailty trajectories in middle-aged and older adults: a longitudinal study. Nutr J. (2025) 24:89. doi: 10.1186/s12937-025-01153-1 40462132 PMC12131399

[27] Guerriero F Reid MC . Linking persistent pain and frailty in older adults. Pain Med. (2020) 21:61–6. doi: 10.1093/pm/pnz174 31343692 PMC7999618

[28] Vetrano DL Palmer K Marengoni A Marzetti E Lattanzio F Roller-Wirnsberger R . Frailty and multimorbidity: a systematic review and meta-analysis. J Gerontol A Biol Sci Med Sci. (2019) 74:659–66. doi: 10.1093/gerona/gly110 29726918

[29] Veronese N Stubbs B Noale M Solmi M Pilotto A Vaona A . Polypharmacy is associated with higher frailty risk in older people: an 8-year longitudinal cohort study. J Am Med Dir Assoc. (2017) 18:624–8. doi: 10.1016/j.jamda.2017.02.009 28396180 PMC5484754

[30] Lu Z Er Y Zhan Y Deng X Jin Y Ye P . Association of frailty status with risk of fall among middle-aged and older adults in China: a nationally representative cohort study. J Nutr Health Aging. (2021) 25:985–92. doi: 10.1007/s12603-021-1655-x 34545918 PMC12929974

[31] Ghignone F van Leeuwen BL Montroni I Huisman MG Somasundar P Cheung KL . The assessment and management of older cancer patients: a SIOG surgical task force survey on surgeons' attitudes. Eur J Surg Oncol. (2016) 42:297–304. doi: 10.1016/j.ejso.2015.12.004 26718329

[32] Vermillion SA Hsu FC Dorrell RD Shen P Clark CJ . Modified frailty index predicts postoperative outcomes in older gastrointestinal cancer patients. J Surg Oncol. (2017) 115:997–1003. doi: 10.1002/jso.24617 28437582 PMC7064809

[33] Li CQ Ma JH Liu ZZ Li J . Role of frailty in predicting postoperative pulmonary complications in older patients undergoing major abdominal surgery: a retrospective cohort study. Korean J Anesthesiol. (2025) 78:547–59. doi: 10.4097/kja.24933 40685166 PMC12665396

[34] Hall DE Arya S Schmid KK Carlson MA Lavedan P Bailey TL . Association of a frailty screening initiative with postoperative survival at 30, 180, and 365 days. JAMA Surg. (2017) 152:233–40. doi: 10.1001/jamasurg.2016.4219 27902826 PMC7180387

