# Supplementary material for: Preoperative frailty risk in older patients with colorectal cancer admitted to the ICU after surgery: prevalence, associated factors, and association with in-hospital outcomes
Source: Front Oncol. 2026 Oct 26;16:1948607. doi: 10.3389/fonc.2026.1948607 (PMC13745358; doi:10.3389/fonc.2026.1948607)
Supplement: Supplementary file 1 [file Table1.docx]

**Supplementary Table S1**

Complete generalized linear model output for total hospital length of stay, ICU length of stay, and total hospitalization cost.

| Variable | Total hospital length of stay | | ICU length of stay | | Total hospitalization cost | |
| --- | --- | --- | --- | --- | --- | --- |
|  | Exp(B) (95% *CI*) | *P* value | Exp(B) (95% *CI*) | *P* value | Exp(B) (95% *CI*) | *P* value |
| Frailty risk | **1.184 (1.096, 1.278)** | **< 0.001** | **1.323 (1.127, 1.554)** | **< 0.001** | **1.115 (1.019, 1.220)** | **0.018** |
| Age | 1.000 (0.994, 1.005) | 0.912 | 0.984 (0.974, 0.995) | 0.005 | 0.995 (0.989, 1.001) | 0.113 |
| ASA (reference: II) | |  |  |  |  |  |
| III | 0.942 (0.848, 1.046) | 0.264 | 1.035 (0.832, 1.288) | 0.757 | 0.988 (0.873, 1.117) | 0.844 |
| IV | 0.932 (0.758, 1.145) | 0.502 | 1.592 (0.987, 2.566) | 0.057 | 0.923 (0.726, 1.173) | 0.512 |
| Surgery type | 1.100 (0.927, 1.305) | 0.276 | 1.829 (1.214, 2.754) | 0.004 | 1.173 (0.962, 1.430) | 0.115 |
| Intraoperative blood transfusion | 1.199 (1.087, 1.323) | < 0.001 | 1.155 (0.943, 1.415) | 0.163 | 1.110 (0.989, 1.245) | 0.076 |
| Operative time | 1.020 (0.989, 1.053) | 0.215 | 0.942 (0.888, 1.000) | 0.048 | 1.041 (1.003, 1.081) | 0.034 |
| TNM stage (reference: 0/I) | |  |  |  |  |  |
| II | 0.968 (0.868, 1.081) | 0.567 | 0.940 (0.752, 1.175) | 0.586 | 1.072 (0.944, 1.218) | 0.284 |
| III | 0.959 (0.854, 1.078) | 0.485 | 0.842 (0.666, 1.064) | 0.150 | 0.948 (0.828, 1.086) | 0.443 |
| IV | 1.031 (0.882, 1.206) | 0.699 | 1.222 (0.885, 1.688) | 0.224 | 0.980 (0.817, 1.176) | 0.827 |
| Nutritional risk | 0.963 (0.891, 1.041) | 0.347 | 1.080 (0.924, 1.262) | 0.335 | 1.071 (0.978, 1.174) | 0.139 |

**Supplementary Table S2**

Multivariable logistic regression analysis of mFI-5 as a continuous variable for postoperative complications.

| Variable | Overall complications | | Severe complications  (Clavien-Dindo grade ≥ III) | |
| --- | --- | --- | --- | --- |
|  | *OR* (95% *CI*) | *P* value | *OR* (95% *CI*) | *P* value |
| Frailty risk | **1.605 (1.243, 2.074)** | **< 0.001** | **1.494 (1.115, 2.001)** | **0.007** |
| Age | 1.001 (0.965, 1.037) | 0.976 | 1.044 (0.999, 1.092) | 0.055 |
| ASA (reference: II) |  | 0.202 |  | 0.287 |
| III | 1.331 (0.674, 2.628) | 0.410 | 0.799 (0.352, 1.815) | 0.592 |
| IV | 3.660 (0.882, 15.182) | 0.074 | 2.027 (0.495, 8.295) | 0.326 |
| Surgery type | 0.869 (0.285, 2.647) | 0.805 | 2.842 (0.887, 9.101) | 0.079 |
| Intraoperative blood transfusion | 1.805 (0.933, 3.496) | 0.080 | 1.881 (0.936, 3.778) | 0.076 |
| Operative time | 1.478 (1.181, 1.850) | < 0.001 | 1.193 (0.940, 1.513) | 0.147 |
| TNM stage (reference: 0/I) | | 0.324 |  | 0.840 |
| II | 0.637 (0.314, 1.293) | 0.212 | 1.035 (0.434, 2.470) | 0.937 |
| III | 1.039 (0.492, 2.197) | 0.920 | 0.844 (0.329, 2.164) | 0.724 |
| IV | 0.968 (0.348, 2.695) | 0.951 | 0.657 (0.180, 2.394) | 0.524 |
| Nutritional risk | 1.452 (0.868, 2.428) | 0.156 | 1.144 (0.617, 2.123) | 0.669 |

**Supplementary Table S3**

Generalized linear model analysis of mFI-5 as a continuous variable for total hospital length of stay, ICU length of stay, and total hospitalization cost.

| Variable | Total hospital length of stay | | ICU length of stay | | Total hospitalization cost | |
| --- | --- | --- | --- | --- | --- | --- |
|  | Exp(B) (95% *CI*) | *P* value | Exp(B) (95% *CI*) | *P* value | Exp(B) (95% *CI*) | *P* value |
| Frailty risk | **1.083 (1.043, 1.124)** | **< 0.001** | **1.189 (1.105, 1.279)** | **< 0.001** | 1.035 (0.991, 1.081) | 0.125 |
| Age | 0.999 (0.994, 1.005) | 0.756 | 0.981 (0.970, 0.992) | < 0.001 | 0.995 (0.989, 1.001) | 0.136 |
| ASA (reference: II) | |  |  |  |  |  |
| III | 0.946 (0.852, 1.051) | 0.301 | 1.053 (0.848, 1.308) | 0.638 | 0.996 (0.880, 1.127) | 0.951 |
| IV | 0.950 (0.774, 1.167) | 0.627 | 1.661 (1.042, 2.648) | 0.033 | 0.949 (0.747, 1.205) | 0.666 |
| Surgery type | 1.108 (0.934, 1.315) | 0.241 | 1.849 (1.237, 2.764) | 0.003 | 1.171 (0.960, 1.430) | 0.120 |
| Intraoperative blood transfusion | 1.197 (1.085, 1.320) | < 0.001 | 1.137 (0.932, 1.388) | 0.205 | 1.106 (0.986, 1.242) | 0.086 |
| Operative time | 1.025 (0.993, 1.057) | 0.128 | 0.958 (0.904, 1.015) | 0.146 | 1.044 (1.005, 1.083) | 0.025 |
| TNM stage (reference: 0/I) | |  |  |  |  |  |
| II | 0.960 (0.860, 1.072) | 0.466 | 0.904 (0.725, 1.127) | 0.371 | 1.072 (0.943, 1.219) | 0.287 |
| III | 0.954 (0.849, 1.072) | 0.428 | 0.806 (0.639, 1.017) | 0.069 | 0.952 (0.831, 1.091) | 0.480 |
| IV | 1.017 (0.869, 1.190) | 0.835 | 1.069 (0.771, 1.481) | 0.690 | 0.983 (0.818, 1.180) | 0.850 |
| Nutritional risk | 0.956 (0.884, 1.034) | 0.262 | 1.097 (0.940, 1.279) | 0.240 | 1.065 (0.972, 1.168) | 0.177 |

**Supplementary Table S4**

Multivariable logistic regression analysis of postoperative complications after excluding patients aged ≥ 80 years.

| Variable | Overall complications | | Severe complications  (Clavien-Dindo grade ≥ III) | |
| --- | --- | --- | --- | --- |
|  | *OR* (95% *CI*) | *P* value | *OR* (95% *CI*) | *P* value |
| Frailty risk | **2.986 (1.638, 5.443)** | **< 0.001** | **2.753 (1.208, 6.276)** | **0.016** |
| Age | 0.981 (0.929, 1.035) | 0.481 | 0.994 (0.927, 1.067) | 0.877 |
| ASA (reference: II) |  | 0.827 |  | 0.906 |
| III | 1.238 (0.574, 2.672) | 0.586 | 0.798 (0.292, 2.176) | 0.659 |
| IV | 1.519 (0.282, 8.182) | 0.626 | 0.785 (0.115, 5.359) | 0.805 |
| Surgery type | 1.377 (0.375, 5.054) | 0.630 | 4.608 (1.153, 18.410) | 0.031 |
| Intraoperative blood transfusion | 2.385 (1.010, 5.632) | 0.047 | 3.366 (1.371, 8.260) | 0.008 |
| Operative time | 1.529 (1.163, 2.009) | 0.002 | 1.064 (0.794, 1.425) | 0.678 |
| TNM stage (reference: 0/I) | | 0.496 |  | 0.849 |
| II | 0.592 (0.255, 1.375) | 0.223 | 1.529 (0.457, 5.118) | 0.491 |
| III | 0.895 (0.374, 2.142) | 0.804 | 1.145 (0.322, 4.077) | 0.834 |
| IV | 0.937 (0.285, 3.079) | 0.914 | 1.526 (0.332, 7.008) | 0.587 |
| Nutritional risk | 1.530 (0.828, 2.825) | 0.174 | 1.097 (0.503, 2.392) | 0.815 |

**Supplementary Table S5**

Generalized linear model analysis of total hospital length of stay, ICU length of stay, and total hospitalization cost after excluding patients aged ≥ 80 years.

| Variable | Total hospital length of stay | | ICU length of stay | | Total hospitalization cost | |
| --- | --- | --- | --- | --- | --- | --- |
|  | Exp(B) (95% *CI*) | *P* value | Exp(B) (95% *CI*) | *P* value | Exp(B) (95% *CI*) | *P* value |
| Frailty risk | **1.249 (1.139, 1.369)** | **< 0.001** | 1.197 (0.989, 1.448) | 0.065 | **1.146 (1.031, 1.274)** | **0.012** |
| Age | 1.000 (0.992, 1.008) | 0.984 | 0.984 (0.968, 1.000) | 0.054 | 0.989 (0.980, 0.998) | 0.019 |
| ASA (reference: II) | |  |  |  |  |  |
| III | 0.887 (0.787, 1.000) | 0.051 | 1.075 (0.837, 1.379) | 0.573 | 0.948 (0.826, 1.088) | 0.446 |
| IV | 0.824 (0.631, 1.075) | 0.153 | 1.984 (1.085, 3.626) | 0.026 | 0.958 (0.708, 1.296) | 0.782 |
| Surgery type | 1.174 (0.950, 1.449) | 0.137 | 1.382 (0.854, 2.234) | 0.188 | 1.250 (0.985, 1.586) | 0.066 |
| Intraoperative blood transfusion | 1.213 (1.068, 1.377) | 0.003 | 1.104 (0.853, 1.430) | 0.452 | 1.068 (0.923, 1.236) | 0.374 |
| Operative time | 1.000 (0.963, 1.038) | 0.993 | 0.927 (0.865, 0.993) | 0.031 | 1.030 (0.986, 1.076) | 0.188 |
| TNM stage (reference: 0/I) | |  |  |  |  |  |
| II | 0.994 (0.872, 1.132) | 0.922 | 1.104 (0.848, 1.437) | 0.464 | 1.160 (1.000, 1.345) | 0.050 |
| III | 0.960 (0.837, 1.101) | 0.556 | 0.963 (0.730, 1.271) | 0.792 | 0.945 (0.808, 1.103) | 0.472 |
| IV | 1.110 (0.923, 1.334) | 0.268 | 1.665 (1.144, 2.424) | 0.008 | 1.061 (0.861, 1.308) | 0.578 |
| Nutritional risk | 0.920 (0.838, 1.009) | 0.077 | 1.042 (0.867, 1.252) | 0.661 | 1.054 (0.947, 1.171) | 0.335 |

Note: In the same sensitivity analysis population, when mFI-5 was modeled as a continuous variable (per 1-point increase), each 1-point increase was significantly associated with longer ICU stay (Exp(B) = 1.150, 95% *CI*: 1.050 ~ 1.260, *P* = 0.003).

**Supplementary Table S6**

Multivariable logistic regression analysis of postoperative complications after excluding patients with unplanned ICU admission

| Variable | Overall complications | | Severe complications  (Clavien-Dindo grade ≥ III) | |
| --- | --- | --- | --- | --- |
|  | *OR* (95% *CI*) | *P* value | *OR* (95% *CI*) | *P* value |
| Frailty risk | **3.053 (1.788, 5.213)** | **< 0.001** | **2.665 (1.303, 5.454)** | **0.007** |
| Age | 1.009 (0.971, 1.048) | 0.648 | 1.056 (1.004, 1.110) | 0.034 |
| ASA (reference: II) |  | 0.767 |  | 0.832 |
| III | 1.313 (0.629, 2.739) | 0.468 | 0.803 (0.310, 2.077) | 0.651 |
| IV | 1.220 (0.237, 6.285) | 0.812 | 1.119 (0.184, 6.797) | 0.902 |
| Surgery type | 0.515 (0.136, 1.949) | 0.328 | 1.622 (0.363, 7.254) | 0.527 |
| Intraoperative blood transfusion | 2.054 (1.014, 4.162) | 0.046 | 2.605 (1.207, 5.621) | 0.015 |
| Operative time | 1.513 (1.184, 1.933) | < 0.001 | 1.193 (0.910, 1.564) | 0.202 |
| TNM stage (reference: 0/I) | | 0.124 |  | 0.887 |
| II | 0.510 (0.239, 1.092) | 0.083 | 0.994 (0.375, 2.638) | 0.991 |
| III | 0.927 (0.418, 2.056) | 0.852 | 0.763 (0.263, 2.215) | 0.619 |
| IV | 1.078 (0.369, 3.146) | 0.890 | 0.722 (0.173, 3.021) | 0.656 |
| Nutritional risk | 1.574 (0.906, 2.734) | 0.107 | 1.058 (0.532, 2.102) | 0.873 |

**Supplementary Table S7**

Generalized linear model analysis of total hospital length of stay, ICU length of stay, and total hospitalization cost after excluding patients with unplanned ICU admission.

| Variable | Total hospital length of stay | | ICU length of stay | | Total hospitalization cost | |
| --- | --- | --- | --- | --- | --- | --- |
|  | Exp(B) (95% *CI*) | *P* value | Exp(B) (95% *CI*) | *P* value | Exp(B) (95% *CI*) | *P* value |
| Frailty risk | **1.141 (1.058, 1.231)** | **< 0.001** | **1.131 (1.022, 1.252)** | **0.017** | **1.154 (1.064, 1.251)** | **< 0.001** |
| Age | 1.001 (0.995, 1.006) | 0.740 | 0.996 (0.989, 1.003) | 0.265 | 1.003 (0.997, 1.009) | 0.377 |
| ASA (reference: II) | |  |  |  |  |  |
| III | 0.958 (0.862, 1.064) | 0.420 | 1.198 (1.041, 1.377) | 0.012 | 0.973 (0.869, 1.089) | 0.629 |
| IV | 0.937 (0.742, 1.183) | 0.585 | 0.883 (0.647, 1.205) | 0.433 | 0.847 (0.659, 1.087) | 0.192 |
| Surgery type | 1.073 (0.893, 1.290) | 0.451 | 1.328 (1.040, 1.695) | 0.023 | 1.154 (0.949, 1.403) | 0.151 |
| Intraoperative blood transfusion | 1.227 (1.111, 1.355) | < 0.001 | 1.036 (0.905, 1.184) | 0.611 | 1.134 (1.020, 1.261) | 0.020 |
| Operative time | 1.039 (1.005, 1.073) | 0.023 | 1.039 (0.993, 1.086) | 0.099 | 1.066 (1.029, 1.104) | < 0.001 |
| TNM stage (reference: 0/I) | |  |  |  |  |  |
| II | 0.950 (0.851, 1.060) | 0.354 | 0.822 (0.711, 0.950) | 0.008 | 1.010 (0.899, 1.135) | 0.868 |
| III | 0.940 (0.836, 1.056) | 0.297 | 0.861 (0.738, 1.004) | 0.056 | 0.941 (0.831, 1.066) | 0.339 |
| IV | 0.970 (0.830, 1.133) | 0.697 | 0.810 (0.658, 0.997) | 0.047 | 1.055 (0.893, 1.245) | 0.530 |
| Nutritional risk | 1.001 (0.925, 1.084) | 0.971 | 1.071 (0.965, 1.190) | 0.197 | 1.016 (0.934, 1.105) | 0.713 |
